# Laparoscopic Sacrohysteropexy for Pelvic Organ Prolapse and the Technique Used for Uterine Preservation: A Case Report

**DOI:** 10.7759/cureus.54989

**Published:** 2024-02-26

**Authors:** Shogo Nishii, Tetsuya Ishikawa, Yoshiyuki Okada, Akihiko Sekizawa

**Affiliations:** 1 Obstetrics and Gynecology, Showa University, Tokyo, JPN; 2 Obstetrics and Gynecology, Female Pelvic Health Center, Showa University Northern Yokohama Hospital, Kanagawa, JPN

**Keywords:** pop-q, laparoscopic technique, pelvic organ prolapse, uterine preservation, laparoscopic sacrohysteropexy

## Abstract

Pelvic organ prolapse (POP) is prevalent among middle-aged and older women, and its prevalence is expected to increase in Japan in the future. Laparoscopic surgery for POP is covered by insurance and is currently a minimally invasive procedure. There are multiple treatment approaches for the uterus, especially sacrohysteropexy, for patients who wish to preserve their uterus. This approach requires an understanding of its anatomical characteristics, including how the arm is threaded. However, specific techniques for uterine preservation have not yet been thoroughly investigated or reported.

Here, we discuss the innovative operative techniques for uterine preservation and mesh application achieved by laparoscopic sacrohysteropexy performed at our hospital. A 34-year-old woman presented at our hospital with a uterine prolapse in the hope of undergoing laparoscopic sacrohysteropexy. The anterior vaginal wall was dissected, the mesh fixed, and the right and left intrauterine foramina next to the cervix were deployed and released. The anterior vaginal wall mesh penetrated the released mesentery and was integrated with the mesh of the posterior vaginal wall. It was fixed to the anterior aspect of the cape angle by using a subperitoneal tunnel. This surgical case is currently under follow-up, with no recurrence to date. We elaborate upon the ingenious insertion site of the port for the preservation of the uterus, the secure fixation of the mesh to the uterus, and the traction method. Unlike laparoscopic sacrocolpopexy and sacrocervicopexy, laparoscopic sacrohysteropexy necessitates at least the aforementioned techniques. At our institution, we perform sacrohysteropexy following the method outlined in this case. A more efficient technique is expected to emerge as larger-scale studies accumulate additional cases, ultimately leading to widespread acceptance and standardization of the approach.

## Introduction

Pelvic organ prolapse (POP) is one of the most common conditions affecting quality of life and occurs mainly in middle-aged and older women. Pelvic organs such as the uterus, rectum, and bladder slip out of their normal positions and protrude into or out of the vagina, significantly worsening the patient’s quality of life. As the Japanese population ages, the incidence of POP is expected to increase. It is more common in individuals who have undergone vaginal delivery and in the older population, and its prevalence and demand for treatment will continue to grow as the Japanese population expands [1]. According to the International Urogynecologic Association and American Urogynecologic Society, uterine management in POP surgery can be divided into three groups: total hysterectomy (sacrocolpopexy), supracervical hysterectomy (sacrocervicopexy), and uterine preservation (sacrohysteropexy). Generally, supracervical hysterectomy (sacrocervicopexy) is performed in Japan [2,3]. However, as the number of patients increases, an increasing demand for uterine preservation (sacrohysteropexy) is expected. Although many studies have compared sacrohysteropexy to sacrocervicopexy [4-6], specific techniques for uterine preservation have not been thoroughly investigated or reported. Here, we discuss the surgical techniques used for laparoscopic sacrohysteropexy in a representative patient at our hospital. This article was presented as an abstract at the 58th meeting of the Japan Society of Gynecologic and Obstetric Endoscopy and Minimally Invasive Therapy on August 4, 2018.

## Case presentation

The patient was a 34-year-old woman with a body mass index of 19.5 kg/m2. She underwent forceps delivery at 30 years of age and a cesarean section at 31 years of age (G2P2). She visited our hospital with the chief complaint of uterine prolapse. According to the POP-Q progression classification, the patient had stage III prolapse [7]. Symptoms of uterine prolapse appeared after delivery; however, despite ring insertion, the prolapse persisted. This case of uterine prolapse, a Delancey [8] level I injury, was determined to be an indication for laparoscopic sacrohysteropexy. The surgery was performed by a technical endoscopist certified by the Japanese Society of Obstetrics and Gynecology and a trainee obstetrician/gynecologist who was also a certified technical endoscopist with three years of experience.

Laparoscopic surgical technique

**Figure 1:**
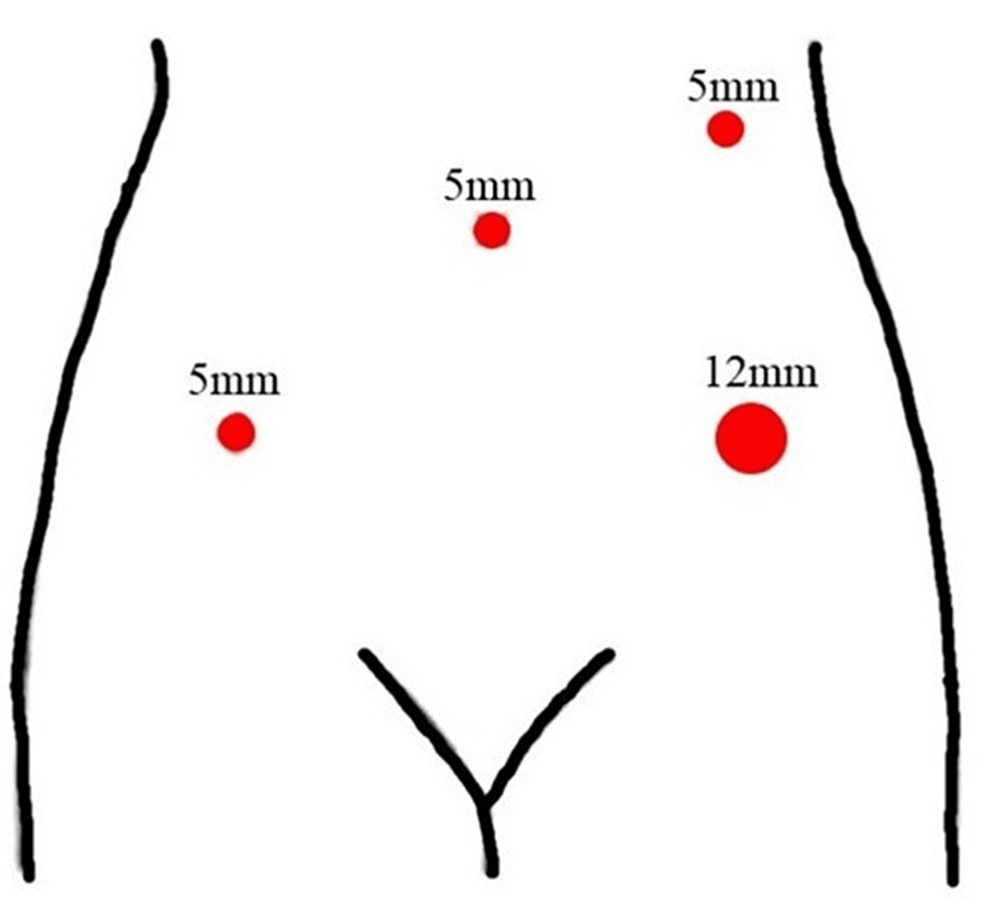
Trocar placement A 5-mm trocar was placed at the umbilicus, a 12-mm trocar at the two medial transverse fingers of the left lateral iliac spine, another 5-mm trocar at the two medial transverse fingers of the right lateral iliac spine, and a final 5-mm trocar at the left midline. Diagram created by author Shogo Nishii.

The operating table was set in a 15-degree head-down position. The insufflation pressure was set at 200 mmHg. Access to the surgical site was achieved via four ports, and device placement was performed in parallel. A 5-mm trocar was placed in the umbilicus, a 12-mm trocar was placed 1 cm to 2 cm medial to the left superior anterior iliac spine, and a 5-mm trocar was placed 1 cm to 2 cm medial to the right superior anterior iliac spine. The last 5-mm trocar was placed in the left midline in the umbilical position (Figure 1). The surgeon positioned the right port (left midline trocar) slightly cephalad, depending on the size of the uterus, as the uterus may be obstructed during dissection on the rectal side.

**Figure 2:**
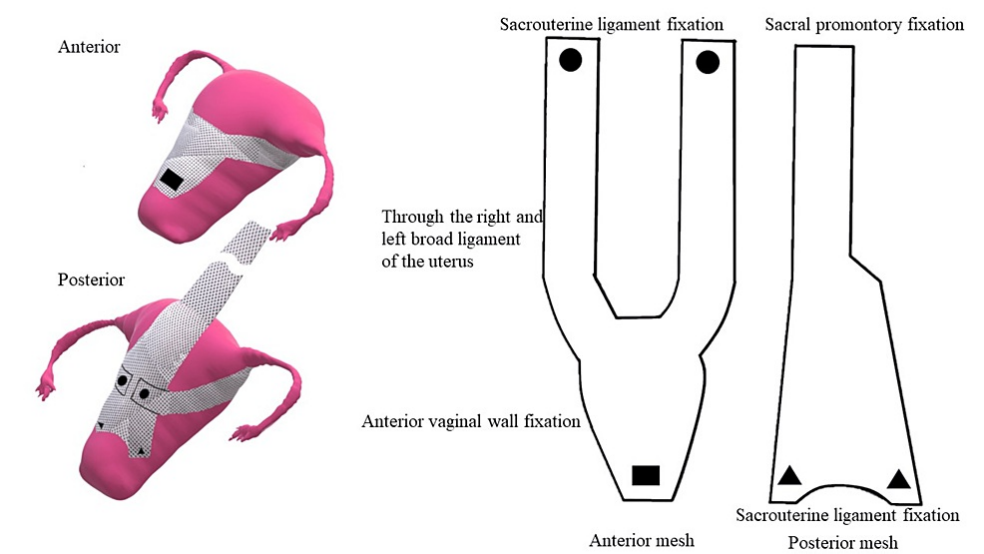
Location of the mesh The left and right mesh legs were threaded through the bilateral interspaces and aligned with the posterior wall of the uterus. Image credit: Author Shogo Nishii

**Figure 3:**
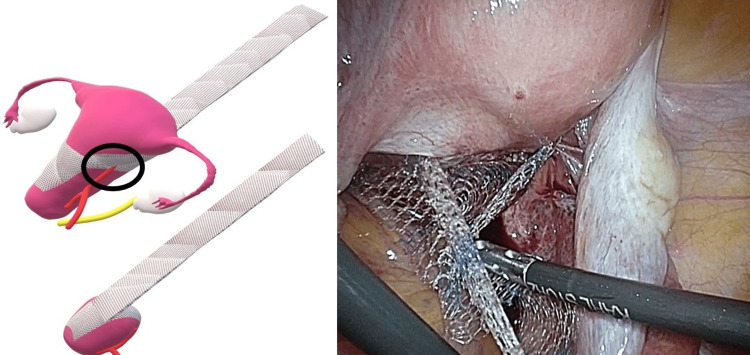
Mesh positioning The anchoring location of the mesh and uterus should be such that the anterior mesh does not exert excessive pressure on the uterine artery. Image credit: Author Shogo Nishii

**Figure 4:**
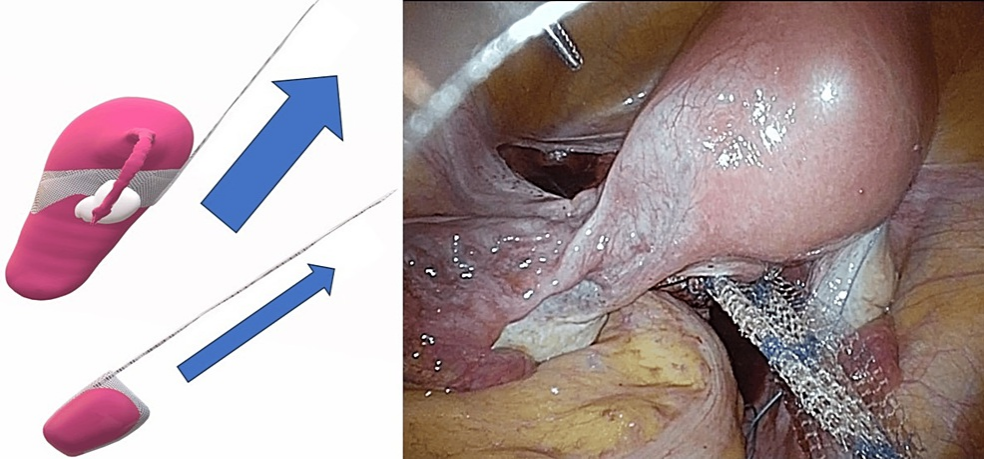
Tow direction Strong traction is required due to the dead weight of the uterus. The traction vector of the mesh is different from that of subtotal hysterectomy and may result in traction in a nonphysiological direction. Image credit: Author Shogo Nishii

Regarding the shape of the mesh, a bipodal anterior mesh was used to penetrate the right and left broad mesenteries (Figure 2). The sigmoid colon was suspended over the left lateral abdominal wall to facilitate visualization. The rectovaginal fascia was dissected to expose the anterior surface of the rectal and anorectal muscles. The posterior mesh was then secured to the anorectalis muscle and sacral uterine ligament using 2-0 Ti-Cron^TM^ (non-absorbable braided thread; Medtronic, Tokyo, Japan) and 3-0 Vicryl^TM^ (absorbable braided thread; Ethicon Inc., Raritan, NJ, USA). Next, the pubocervical fascia was dissected, and the anterior mesh was secured to the vaginal wall near the bladder neck and uterine cervix with 2-0 Tycron and 3-0 Vicryl, respectively. The right and left mesh legs were passed through the broad ligament of the uterus, ensuring that the anterior mesh did not excessively compress the uterine artery (Figure 3). The anterior aspect of the sacral promontory angle was deployed, and the anterior and posterior mesh legs were secured to the anterior longitudinal ligament using a 1-Tefdesser II^TM^ (Crownjun Kono Co. Ltd., Tokyo, Japan) (Figure 4). Finally, the retroperitoneum was closed to complete the surgery. Postoperatively, the patient was followed up after two months, six months, one year, two years, and three years as an outpatient, and physical findings were evaluated using the POP questionnaire (POP-Q).

Outcomes and follow-up

The total operative time for the patient was 150 minutes, and the total blood loss was 215 ml. No intraoperative injuries to other organs, such as the bladder or intestinal tract, were observed. The postoperative course was uneventful, and the patient was discharged on postoperative day 5. Neither subjective nor objective recurrence was observed after five years. The patient originally had no menstrual problems. And no menstrual problems after surgery have been observed to date.

## Discussion

Various surgical techniques are widely available for treating POP; however, selecting the correct technique can be challenging. Although the usefulness of open sacrovaginal fusion (abdominal sacrocolpopexy) has been previously reported [8], recent advancements in laparoscopic surgical techniques have demonstrated the advantages of a minimally invasive laparoscopic approach over the open approach [9]. The number of patients undergoing laparoscopic surgery has increased dramatically since the introduction of insurance coverage for laparoscopic surgery in Japan in 2014. Our hospital first introduced laparoscopic surgery in 2014 based on Ross’s method [10]. The formation of a retroperitoneal tunnel reduces the number of sutures in contact with the intestinal tract, thereby decreasing the incidence of intestinal complications. Although surgical techniques vary among institutions, the uterus is primarily treated using three major approaches: total hysterectomy (sacrocolpopexy), supracervical hysterectomy (sacrocervicopexy), and uterine preservation (sacrohysteropexy). Because of the potential loss of sexual function and the reported increased rate of mesh exposure [11], combined total hysterectomy is not performed at our institution unless abnormal uterine findings, such as cytological findings, are observed. Sacrocervicopexy is straightforward: the uterine body is removed, the pelvic cavity is widely expanded, and the cervical mesh is aligned from the cervix to the sacrum.

However, sacrohysteropexy requires two legs of the anterior mesh to bypass the right and left pia mater and fix the uterus to the sacrum. It is considered relatively minimally invasive because it does not involve any uterine incision and is associated with less operative time and blood loss than sacrocervicopexy [12]. The advantage of this technique is the preservation of supporting tissues, such as the sacrouterine and basal ligaments, which support the cervix. However, because the anterior mesh is wedged into the retroperitoneum and the uterus is left behind during posterior vaginal wall dissection, we do not believe that the operative time can generally be reduced.

**Figure 5:**
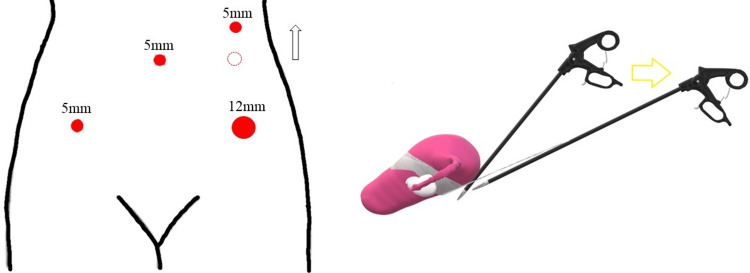
Port location per our sacrohysteropexy technique Surgeons should place the right forceps port as cephalad as possible. Image credit: Author Shogo Nishii

We propose adapting the modifications to the sacrohysteropexy technique performed at our hospital. First, parallel port placement is recommended because of interference between the surgeon’s right-hand forceps and the uterine body. In the case of diamond placement, the right forceps should be inserted from the head as much as possible, and the uterus should be lifted by a direct needle to the abdominal wall (Figure 5). The position of the uterus influences operative time. Moreover, the fixation and position of the mesh should be fixed extensively because the weight of the uterus requires a stronger traction force than in the case of supracervical hysterectomy. Because the traction vector of the mesh will be different from that of supracervical hysterectomy and the traction may be in an unphysiological direction, it is necessary to be aware that the mesh fixation site is also different than in supracervical hysterectomy. Furthermore, the anchoring locations of the mesh and uterus must be devised so that the anterior mesh does not excessively compress the uterine artery.

Although varying recurrence and success rates have been reported [2-4], this technique is widely used among institutions but has not been well documented. A limitation of this case study was that the port location, mesh traction vector, and mesh fixation site were not quantified. We believe that it is necessary to accumulate more cases and investigate the long-term prognoses to obtain more generalizable results.

## Conclusions

Our sacrohysteropexy technique involves an ingenious port insertion site for preserving the uterus, ensuring secure fixation of the mesh to the uterus, and employing the traction method. In contrast to the techniques employed in laparoscopic sacrocolpopexy or sacrocervicopexy, laparoscopic sacrohysteropexy necessitates the inclusion of the aforementioned techniques at a minimum. Further investigation is needed into sacrohysteropexy techniques and their associated benefits, including long-term prognosis.
